# Preventing Harmful Internet Use-Related Addiction Problems in Europe: A Literature Review and Policy Options

**DOI:** 10.3390/ijerph17113797

**Published:** 2020-05-27

**Authors:** Olatz Lopez-Fernandez, Daria J. Kuss

**Affiliations:** 1 Monash Addiction Research Centre, Turning Point, Easter Health Clinical School, Monash University, Clayton, VIC 3800, Australia; 2 International Gaming Research Unit, Cyberpsychology Research Group, Psychology Department, Nottingham Trent University, Nottingham NG1 4FQ, UK; Daria.Kuss@ntu.ac.uk

**Keywords:** Internet addiction, problematic Internet use, generalized Internet addiction, online gaming addiction, online gambling addiction, Europe, policy option, prevention, public health

## Abstract

Internet use-related addiction problems are increasingly being recognized on a European scale due to international health organizations considering gaming addiction. In April 2013, the American Psychiatric Association recognized Internet Gaming Disorder in the fifth Diagnostic and Statistical Manual of Mental Disorders, and in April 2018, the World Health Organization included Gaming Disorder in the eleventh International Classification of Diseases. However, findings on these problems within this period are lacking in Europe, and a preventive approach is missing globally. A detailed critical literature review was conducted using PsycINFO and Web of Science in this five-year period. A total of 19 studies were reviewed and problems identified were: generalized Internet addiction and online gaming and gambling addictions across seven European countries (i.e., Spain, Germany, France, Italy, Greece, The Netherlands, and Denmark). The individuals with problematic use were found to be educated adolescents, usually young males with comorbid disorders, and gaming and gambling disorders were implicated in the most severe cases. Cognitive behavioral therapy was the main treatment, sometimes combined with a systemic approach for adolescents. Prevalence, high-risk populations, and factors contributing to these addiction problems are discussed, and a set of policy options are developed for this region. The implications for early detection, diagnosis, treatment, and prevention in Europe are considered.

## 1. Introduction

Contemporary use of the Internet has led to a number of benefits in the health field (e.g., digital health), but also negative impacts at an individual and psychological level (e.g., gaming addiction).

Excessive Internet use has been classed in the mid-nineties as Internet Addiction (IA) [1], Problematic Internet Use (PIU) [2], or as technological (behavioral) addiction [3]. This broad term, however, has evolved and at present encompasses many types of addiction problems related to generalized Internet addiction (GIA) and a set of specific addictive uses of the Internet [4]. These include online gambling, online gaming, social networking, and cybersex, which are the most prevalent ones that have evolved alongside gaming addiction [5]. These behavioral problems can be engaged in using any device as the Internet is ubiquitous. Accordingly, during the last decade, the Internet has facilitated the development of addiction problems through online technology in many ways, and is associated with health problems (e.g., distress, functional impairment, and comorbidity [6]).

During the last decade, international health bodies, which publish diagnostic manuals for mental health diseases, recognized two associated conditions as behavioral addictions, i.e., gambling and gaming disorders. First, the American Psychiatric Association (APA) proposed Internet Gaming Disorder (IGD) in its fifth Diagnostic and Statistical Manual of Mental Disorders (DSM-5) within its third appendix in April 2013 [7]. Subsequently, the World Health Organization (WHO) included Gaming Disorder (GD) in its first version of the eleventh International Classification of Diseases (ICD-11) in April 2018 [8]. This inclusion has produced the following consequences. Firstly, gambling and gaming disorders have been recognized in the mental health sciences and by health practitioners as behavioral or process addictions, leading to many debates [9,10]. Secondly, the mass media have alerted the general public regarding these emerging online addiction problems which usually affect young populations [11,12]. Thirdly, these addictions are now understood as international public health concern, and the preventive actions undertaken have had limited success and focused on English speaking countries (i.e., in American, European, and Australasian regions [13]), and Asian countries [14].

However, to the authors’ knowledge, no literature review has been conducted focusing on the period when gaming addiction was officially recognized by global health organizations, within an intercultural continental region (i.e., Europe), to detect the main concerns, and to propose a set of policy options which are culturally and geographically based. For these reasons, the European Parliament’s Scientific Foresight Unit (STOA) endeavored to perform a recent literature review to study the individual and psychological aspects of the harms associated with Internet use, including IA and related harms (e.g., gaming addiction) in the European Union (EU) [15].

To the authors’ knowledge, only two reviews exist with similar characteristics, but both with an international scope rather than a regional focus performed in 2016 [6,16].

Kuss and Lopez-Fernandez [6] focused on clinical research on IA and reported characteristics of treatment seekers and online addiction treatments. First, treatment seeker characteristics from various continents included European clinical studies performed in Germany, The Netherlands, and Greece, and focused on both, GIA and gaming addiction problems (among other comorbidities). Second, psychopharmacotherapy was covered, which appeared to have positive effects in decreasing IA symptomatology and Internet gaming addiction problems through antidepressants and anxiolytics, and obsessive–compulsive disorders (OCD) and attention deficit hyperactivity disorder (ADHD) medications for comorbid problems. Third, psychological therapies usually with an individual approach (e.g., cognitive behavioral therapy (CBT)) were applied to outpatients, apart from a few group therapy approaches (e.g., multi-family group therapy (MFGT)). Fourth, combined treatments were researched, which included psychological treatment in combination with pharmacotherapy or electroacupuncture therapy.

Vondráčková and Gabrhelík’s review [16] focused on IA prevention. First, they stated some target groups may benefit from prevention (e.g., children and adolescents) when it is indicated (e.g., focusing on psychopathological factors). Second, the need to improve specific skills with the help of professionals and other significant individuals (i.e., counsellors, parents) was emphasized. Third, program characteristics were deemed relevant (e.g., information-provision versus interactive interventions). Fourth, environmental interventions were indicated as being needed in some regions (e.g., in countries in which IA is a public health concern where regulation should be promoted, similar to the approach taken by the Chinese government [17]).

Apart from these reviews, a world-wide meta-analysis on IA performed by Chen and Li in 2014 [18] indicated that the global estimated prevalence rate was approximately 6%, with the lowest numbers found in Northern and Western Europe (2.6%). IA prevalence was inversely associated with self-perception of quality of life regarding subjective (e.g., life satisfaction) and objective indicators (e.g., environmental conditions). Furthermore, many cross-cultural studies on IA have emerged since 2012, especially in intercultural regions, such as Europe [19,20]. These studies were school based with adolescent samples and found between 1%–4% estimated prevalence of GIA (which was higher in males). There has been a continuing increase in the number of these studies in the field [21], including mostly cross-national intercontinental studies (covering Asia, America, and Europe), which have researched GIA and estimated its prevalence with psychometric scales, obtaining higher rates in Asian countries and in young male users.

Considering the above, the objective of the present paper was to present a timely critical review of the literature on Internet use-related addiction and associated problems published in Europe between April 2013 (i.e., when IGD was included in the DSM-5′s III appendix) and April 2018 (i.e., when GD was first officially recognized in the ICD-11 beta test version). The aims were to critically analyze online harms by addressing: (i) the cross-cultural approach adopted within the EU, (ii) the users’ characteristics based on community and clinical populations, (iii) Internet use-related addiction problems and the interventions to target the resultant harms in Europe, and (iv) its implications at a public health level with an eye towards prevention. Furthermore, we aim to provide the first set of policy options for harm minimization at the level of the individual in Europe.

## 2. Materials and Methods

A literature review was conducted using the databases PsycINFO and Web of Science between January and April 2018 at Nottingham Trent University (United Kingdom). The rationale to select these two scientific databases was to contain research in Psychology and related disciplines [15]. Initially, PubMed was also selected but the results almost duplicated all outcomes collected via the first two databases, and consequently this search was discarded. PsycINFO and Web of Science are also among the most relevant in the field of Internet addiction covering the majority of current scientific sources targeted in the present paper’s aims. Moreover, they offered sufficient information to perform a timely, expeditious, and recent review, and are among the databases which are usually used in literature reviews published in this field from a disciplinary perspective (i.e., PsycINFO) and also using interdisciplinary approaches (i.e., Web of Science), which allowed us to study the individual and psychological aspects of the harms associated with IA.

The review comprised scientific papers published between April 2013 and April 2018 as this is the period between the official recognition of IGD and GD. The following search was undertaken using the following terms, clusters, and Boolean operators: (“Internet” OR “online” OR “game*” OR “gaming” OR “video gam*” OR “videogame*” OR “video-game*” OR “social network*” OR “social media”) AND (“Addict*” OR “compuls*” OR “problem*” OR “disorder” OR “pathology*” OR “excess*”) AND (“clinic*” OR “treat*” OR “therap*” OR “harm*” OR “risk factor” OR “prevent*”). The search was performed by paper titles as this was the only option available across both search engines.

The inclusion criteria were for studies to: (i) contain empirical data (i.e., data collected using quantitative, qualitative, and mixed methods approaches), (ii) assess online addictions in the EU, (iii) be published between 2013–2018, (iv) include community and clinical samples, (v) provide a full-text article, and (vi) be published in the languages the authors manage (i.e., English, Spanish, French, German, Polish, Italian, and Portuguese).

**Figure 1:**
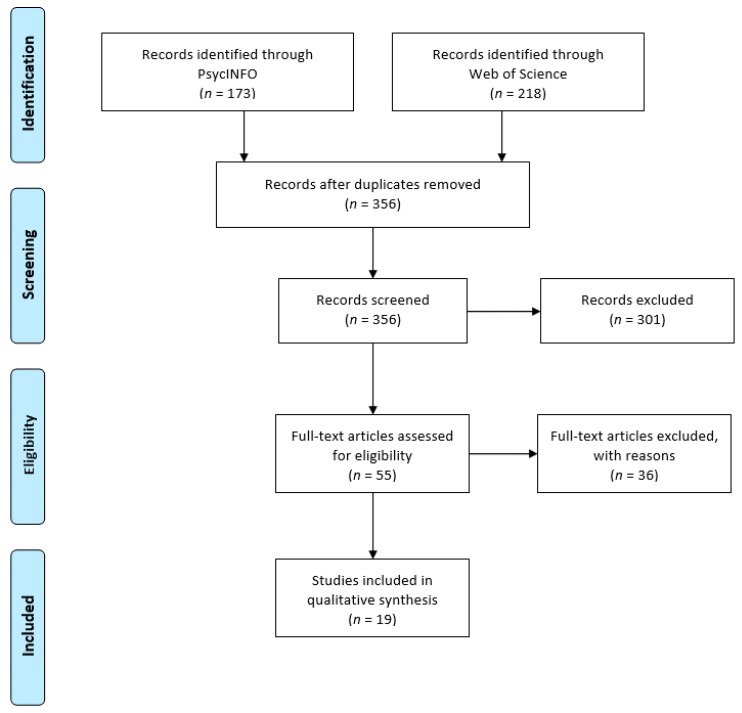
PRISMA Flow diagram of study selection processes.

The literature review was performed as indicated in Figure 1 [22]. Over 390 sources resulted from the initial search. Of these, a great number were filtered out based on the following criteria: (i) duplicates, (ii) meeting and conference abstracts and non-empirical studies (e.g., case studies, anecdotal studies, reviews, editorials, letters, and commentaries), (iii) studies that did not assess IA and related harms in the EU, (iv) studies that were not published between April 2013 and April 2018 (both months included), (v) did not include the population groups targeted (i.e., community and clinical samples), (vi) did not provide a full text article, (vii) were not published in a language the authors manage. Thus, after removing duplicates (*n* = 34), articles in other languages (*n* = 32), conference abstracts (*n* = 188), non-empirical studies (*n* = 81) and non-EU papers (*n* = 36), 19 relevant sources were included in the final analysis.

Two rounds of searches were used: a first round (in January and February 2018) using PsycINFO and subsequently Web of Science, followed by a second round (in April 2018) to ensure all papers were consistently collected and no new paper was published within the specified period and in accordance with the inclusion and exclusion criteria. From the initial pool of 390 papers, after deleting duplicate papers, the remaining 324 results were manually scanned (i.e., title, abstract, key words, and, the paper) to identify the relevant outcomes. Thus, the literature search provided non-exclusive categories of Internet use-related addiction problems as follows: eight Internet addiction papers (i.e., seven by Internet addiction itself, and one including Internet addiction and gaming addiction), 11 online gaming addiction papers (i.e., eight with gaming addiction by itself, and two about gambling and gaming addictions together, and one including Internet addiction and gaming addiction), and three online gambling addiction papers (i.e., own with gambling addiction by itself, and two including gambling and gaming addictions together).

## 3. Results

Data were initially organized into four main categories which emerged in the qualitative analysis of the 19 European empirical papers undertaken by the two co-authors by categories (see Table 1).

Therefore, both authors independently first qualitatively analyzed the papers divided by categories (i.e., O.L.-F. performed the examination of gaming and gambling addiction articles [24,28,30,31,33,34,35,36,38,39,40]; and D.J.K. evaluated Internet addiction articles [23,25,26,27,29,32,37,41]). Therefore, both authors reviewed the contents of the identified articles according to the following categories of analysis: title and journal, authors and country, sample, design, aim(s), measures, results, implications for policy options and prevention, and conclusions for harm minimization [15]. From this categorical analysis the authors proceeded to discuss the main preliminary results, and extracted the information based on the aims with the final purpose of creating a set of preliminary policy options and preventive actions for IA and related harms in Europe. This process included several rounds until theoretical saturation of the contents from all 19 papers was achieved, according to the aims.

The present qualitative and narrative analysis resulted in the division of identified research papers into four categories: (i) the characteristics of problem users (including community and clinical samples); (ii) GIA; (iii) specific IA problems (i.e., gaming, and gambling addictions); and (iv) policy options for preventing Internet use-related harm in Europe. The first category about users’ characteristics is subdivided by Internet use-relate addiction problems (i.e., GIA and specific problems), as the literature shows there are differences in Internet users based on typology of disordered behaviour. Therefore, the categories related to GIA and specific problems were analyzed in detail covering both non-clinical and clinical studies, which were researched from a policy implications perspective. Lastly, the fourth category was divided into respective policy options to reduce Internet harm from an individual person perspective.

Regarding geographical location, half of the studies included in this review (*n* = 10; 53%) were from the Southern European region (countries are ordered from higher to lower frequency): Spain (*n* = 7), Italy (*n* = 2), and Greece (*n* = 1); and 42% (*n* = 8) were from the Western European region: Germany (*n* = 4), France (*n* = 3), and The Netherlands (*n* = 1). Finally, only one study (5%) was conducted in the Northern region (i.e., Denmark).

### 3.1. The Characteristics of Targeted Problem Internet Users

Almost all participants included in the studies were adolescents and young adults from high schools or universities, and the assessed studies dealt with GIA and gaming addiction. Only a few studies assessed online gambling addiction, with participants usually being middle-aged male adults.

#### 3.1.1. Generalized Internet Addiction Users

The main characteristics extracted were:

- Sample sizes: variability depending on the method applied (all research methods were used);
- Age groups: majority of adolescents, and some adults;
- Gender: balanced in adolescent community samples, and more males in clinical samples;
- Regions: In Western and Southern Europe (i.e., Germany, France, Greece, Italy, and Spain).

Sample sizes included studies which varied in number depending on the research methods applied. For instance, samples ranged from 16 Italian Internet-addicted patients investigated through an experiment with a control group to assess the biological causes of IA [32] to a survey with 1,019 German adults to test a new model for GIA [25]. Regarding life stage, participants were usually adolescents and students in high schools [23,27,29], although a few studies included adults [25]. Regarding participant gender, studies on GIA tended to cover both genders in a balanced way. However, when participants were university students, there tended to be more females than males in the sample [25,26,29], and there were significantly more males in clinical samples [27,37]). No study analyzed potential differences between female and male problem Internet users.

#### 3.1.2. Specific Internet Addiction Problem Users: Gamers and Gamblers

The main characteristics extracted from Internet-addicted gamers and gamblers were:

- Sample sizes ranged from one case study to group surveys including mixed methods studies;
- Age groups: majority of adolescents, and a few young adults who were gamers and only adult gamblers;
- Gender: more males, especially in clinical samples and gambling studies;
- Regions: all regions studied (i.e., Spain, France, Germany, The Netherlands, and Denmark).

Sample sizes included clinical case studies of one [38] and nine adolescent patients [24], and online surveys with gaming or gambling participants [30,31] used mixed methods studies combining interviews and surveys on Internet gambling [33,34]. Almost all studies about gaming addiction used adolescent samples [28,38,39,40], and the clinical studies were conducted with males who usually played Massively Multiplayer Online Role-Playing Games (MMORPGs [24,38,39]) and sometimes Multiplayer Online Battle Arena games (MOBA [39]) or First-Person Shooters (FPS [39]). Interestingly, these studies usually included family members (e.g., the mother, both parents, or a sibling [24,28,38,39]), type of family [41], or type of parenting style [28] to treat existing conflicts (e.g., loneliness and discussions with parents) and measured the impact of environmental factors and interventions [28,38,39,40].

However, studies on Internet gambling were conducted with patients within a pathological gambling unit [31,34] and explored factors related to IGD, and some participants were invited from an online gambling site (i.e., Winnimax [33]). These studies came from Spain [31,33,34,35,36,39], France [33,38], Germany [28,41], The Netherlands [40], and Denmark [30].

### 3.2. Generalised Internet Addiction Problems

Eight studies (42.1%) assessed GIA, and referred to non-specific Internet use (i.e., not reliant on the engagement with a particular online activity), and few considered prevention of IA [23]. The studies reviewed were from Germany [25,37], France [26], Greece [27], Italy [23,32], and Spain [29].

Findings suggested a wide range of problems could arise from overusing (e.g., difficulty cutting down, lack of sleep, fatigue, irritability, apathy, racing thoughts, declining grades or poor job performance, and neglecting other duties). Thus, the presence of addiction symptoms (e.g., tolerance), impairment in daily functioning, high comorbidity (i.e., anxiety, depression, and OCD), and risk factors (e.g., preoccupied and fearful attachment styles) were identified. Specifically, problem users tended to present psychological characteristics and co-occurring disorders (i.e., when two or more health problems occur at the same time, e.g., an addiction problem and a mental health disorder are present simultaneously). Usually, these other mental health disorders related to personality disorders, mood disorders, or anxiety disorders. For example, those who were affected by GIA also presented with poor coping strategies and low self-esteem [25], and attachment difficulties (e.g., preoccupied and fearful types [26]). Regarding co-occurring disorders, it seems at least half of the samples presented at least two problems [25,27]). The most prevalent associated problems were depression and anxiety disorder (e.g., the latter with the social subtype [25]). However, CBT emerged as effective in leading to significant changes in symptom experience.

The main characteristics of individuals with GIA in Europe were:

- Almost all studies used Young’s psychometric tests, and their derivatives, whilst finding higher prevalence in clinical studies;
- Models in IA explain risk factors, which are diverse (cognitive, attachment styles, and comorbidity);
- Peer education programs when used as school interventions have good outcomes;
- Clinical interventions rely on cognitive and emotional components, including CBT approaches.

Prevalence rates were higher in clinical studies than in community studies. For instance, Müller et al. [37] estimated a prevalence of 71% of German treatment seekers with the clinical diagnosis of IA; while Andrisano and colleagues [23] found a prevalence rate of 4% of severe Internet-addicted Italian adolescent users among the community sample they studied, which was similar to the other community samples with young Spanish adults, where the prevalence was 10% according to Gonzalez and Orgaz [29]. The scale that most frequently used to measure IA [23,25,27] was the Internet Addiction Test (IAT [42]) and its short version (s-IAT [43]). However, other valid measures have also been used [27,29] (e.g., Online Cognitions Scale (OCS [44]); Index of Problematic Online Experiences (I-POE [45]), and the Assessment of Internet and Computer game Addiction—Scale (AICA-S [41])).

Brandt et al.’s [25] model on GIA explained 64% of GIA variance based on addiction symptoms, and included associated disorders and IA symptoms experience, suggesting users’ cognitions (e.g., poor coping and cognitive expectations) increase the risk of IA. However, comorbidity can also mediate the relationship between symptomatology and factors which seem to act as a cause. Similarly, Danet and Miljkovitch [26] stated fearful and preoccupied attachments can be associated with IA, and Lai et al. [32] suggested a generalized impairment in emotional and cognitive processing abilities in those who suffer from IA, which can be linked to dissociative symptoms. Comorbidities in IA seem, therefore, to be diverse and present in half of Internet-addicted patients [25,27,37,41]. The identified comorbidities include depression, social anxiety, and associated symptoms experienced, such as low self-esteem, low self-efficacy, and high stress vulnerability. According to Müller et al. [27], the majority of treatment seekers present criteria sufficient to be diagnosed with IA, and half of them have comorbidities (i.e., depression, OCD, and dissociative symptoms) and stress. In general, comorbidities include Axis I diagnoses, such as:

- Anxiety Disorders (e.g., panic, social anxiety, and post-traumatic stress disorders);
- Mood Disorders (e.g., major depression, bipolar disorder);
- Eating Disorders (e.g., anorexia nervosa, bulimia nervosa);
- Psychotic Disorders;
- Dissociative Disorders;
- Substance Use Disorders (i.e., drug addictions).

Furthermore, it seems anxiety disorders are associated with the onset of GIA, and mood disorders can be precursors of or follow IA [27].

School interventions which have shown excellent outcomes are the peer education program evaluated by Andrisano and colleagues [23] in Italy, which included brainstorming and video co-creation. In Spain, potential Internet-addicted students [29] also presented with the problem, and this was associated with environmental factors (e.g., family, friends, online interactions, etc.). Both school-based studies came from Southern Europe, suggesting there is a need of educational policies to prevent GIA and related harms in this European region.

Clinical interventions usually aimed to validate tools and cut-off points to estimate the prevalence of GIA [37,41] (e.g., AICA-S [41]).

### 3.3. Specific Internet Addiction Problems: Gaming and Gambling

Twelve papers (63.2%) reported results on online gaming and gambling addictions, nine of which focused only on gaming (44.4%). Thus, these two problems together were more prevalent in comparison with GIA in the assessed samples of European studies.

#### 3.3.1. Internet Gaming Addiction

The main characteristics of Internet gaming addiction in Europe were:

- All studies used different scales and methods (from qualitative to experiments) and addressed prevalence;
- A few models and risk factors emerged (i.e., cognitive, emotional, environmental, and comorbidity);
- Peer education programs used as school interventions have shown contradictory outcomes;
- Clinical studies relied on cognitive, emotional, and personality components and used CBT.

Studies that screened for gaming addiction in community samples were a minority in this section, and usually measured self-perceived problematic video gaming through different devices (e.g., computers and consoles) to assess both offline and online gaming through cross-sectional surveys in Denmark and Spain [30,35]. Regarding their commonalities, males and older adolescents were at a higher risk of gaming addiction problems, non-clinical measures were useful as preventive actions, and programs to reduce gaming were usually effective, and even more so if personality traits (such as impulsivity) were addressed in the interventions.

However, clinical studies were the most common in the samples that were included in the present review, coming from Spain [24,36,39], France [38], The Netherlands [40], and Germany [28]. Patients were brought to health centers by their families, usually by their mother or a sibling [24,38], and in general parent supervision was required [28]. The main factors associated with problematic MMORPG, MOBA and FPS behaviors were dissociation (i.e., a psychological mechanism of stepping out of oneself to be protected from external harm; e.g., bullying or the loss of a loved one), entertainment (e.g., enjoyment and escapism), and virtual friendship (e.g., social relationships in game without any need to personally know one’s fellow gamers; the ‘clan’ or the ‘guild’) [24]. Therefore, there was a need to assess present motivations [38]: to change (e.g., if you continue gaming like this during a decade, what will happen to you?), and to work therapeutically (e.g., playing time was double the usual adult working time per week). Simultaneously, functional analyses were performed (e.g., to support the patient to treat themselves regarding the co-occurring disorders associated with gaming), treating the gaming behaviour (i.e., psychological gaming experience), while addressing alternative pastime opportunities, and improving other relationships.

In CBT interventions, the emotional component was as relevant as the cognitive component; e.g., using techniques related to empathy, self-esteem, self-control, assertiveness, communication skills, or insight [39]. One of the main aspects in the therapeutic intervention was relapse prevention [24,38,39]. Furthermore, one study [28,38,39,40] showed that nonspecific psychiatric disorders pose an increased risk for gaming addiction. This supports the argument that Internet gaming addiction might be a discrete psychiatric entity usually combined with emotional and social problems [24,38]. It can be related to ADHD, Asperger’s, Autism, and other disorders, such as anxiety and depression, social phobia, pervasive developmental disorders, among other comorbid conditions and problems (e.g., parent–child relationship problems, school relationship problems, obesity, cannabis use, and anhedonia). The prognosis is generally positive at three or six months of treatment [24,36,38] for those patients with an externalized profile (i.e., disruptive behaviour disorder, ADHD, and adaptive disorder) or an internalized profile (i.e., anxiety, mood and personality disorders, social relationship problems, previous mental disorders family histories, and individuals who use gaming to escape discomfort experienced in their daily lives [39]). Furthermore, clinicians have stated that increasing numbers of patients sought help through families in the recent years in European public hospitals and health centers [34,36].

Thus, gaming addiction in Europe during the last decade has required both, the development of new short non-clinical measures to screen for it in young adolescents (e.g., using the computer gaming index, console gaming index, or the Internet use index [30]), while clinical studies usually were case studies and used mixed methods dealing with interventions through tailored CBT, including for instance the ‘Individualized Psychotherapeutic Program for the Addiction to the Information and Communication Technologies’ (PIPATIC [39]), and new psychometric tools (e.g., Clinical Video game Addiction test second version(C-VAT 2.0 [40]), or Assessment of Internet and Computer Game Addiction (AICA-S [41])). Related to prevalence, according to Martin-Fernandez et al. [36], 69% of Spanish adolescent patients met the DSM-5 criteria for IGD, and 91% of young Dutch patients met the IGD criteria through the C-VAT 0.2 [40]. However, only 37% of gamblers also experienced video game addiction as co-occurring disorder.

Furthermore, the effectiveness of impulsivity techniques to prevent gaming addiction has been demonstrated [35]. The most studied gaming problem is related to using MMORPGs [24,38,39], which has been researched through qualitative and mixed methods approaches to create a theoretical model [24] or to test CBT interventions [38,39]. The MMORPG online gaming addiction phenomenon has been described by Beranuy et al. [24], including use motivations (e.g., entertainment, escapism or disassociation, and virtual friendship) and factors associated with it, its symptomatology, and consequences (e.g., game context, conflict, and loss of control, respectively). Taquet and Hautekeete [38] and Torres-Rodriguez et al. [39] also highlighted good knowledge of the world of video games by the therapist and a balance between emotional and cognitive components in the intervention are positive factors to ensure therapeutic alliance and successful treatment outcome.

The scales used to measure gaming addiction in this European review were diverse and validated in different languages. These instruments include the AICA-S [41], Assessment of Pathological Computer Gaming (CSV-S [46]), Problem Video Game Playing Scale (PVP [47]), and the Video game dependency test (TDV [35]). Consequently, only a few of the assessed studies measured IGD, as stated by the APA [36,39,40] (e.g., the C-VAT 2.0 [40], the Internet Gaming Disorder test with 20 items (IGD-20 [48])).

School interventions have also been studied [30,35], usually to develop non-clinical measures for problematic gaming and Internet use, screen time, and other problems. The study from Denmark [30] did not find any problems regarding GIA or specific Internet uses in their population of study. On the other hand, a similar Spanish study [35] used an intervention for adolescents to prevent video gaming addiction and to treat two intervention groups with a program to prevent addiction to technologies (i.e., “PrevTec 3.1”). In one group, impulsivity management techniques were added to intensify the positive outcomes of the preventive program, in addition to a waitlist control group. They found the preventive program significantly reduced perceived dependence on video games, and the group who received instructions on impulsivity techniques maintained the successful results in the follow-up better than those who did not receive these techniques in the program. Accordingly, personality traits, such as impulsivity, appear to play a role in prevention on a long-term basis.

Ten lessons have been extracted regarding the problem gamer profile in Europe:

1. It appears they are high-school students,
2. Usually males,
3. Who usually play MMORPGs,
4. Spend considerable time at home alone and game for many hours daily,
5. Treatment is usually sought by parents,
6. These patients present distinct addiction symptomatology,
7. With specific comorbidities (internalized versus externalized profiles),
8. Together with problems with social relationships (e.g., social phobia),
9. CBT has positive results after three and six months, which are maintained after six months,
10. Prognosis improves if family support the treatment.

These studies also highlighted that preventative programs are effective over time in reducing gaming. However, in clinical settings, time spent gaming, age and gender, type of games (e.g., MMORPGs), and type of comorbidities were associated with gaming addiction (e.g., individuals with externalizing profiles have the best prognosis after three months and both profiles have a good prognosis at six months of treatment [36]). Moreover, lack of external parental control should be considered as important risk factor. However, one study did not find any specific psychiatric disorder as a risk factor for this addiction problem [28]. Thus, gaming addiction appears to be a unique clinical entity that can be treated by CBT (e.g., with a treatment length of three to six months). Follow-up studies are required to verify its benefits across groups and cultures.

#### 3.3.2. Internet Gambling Addiction

The main characteristics of Internet gambling addiction in Europe were:

- All studies used different measures for gambling addiction;
- Risk factors emerged, especially when related to gaming addiction;
- Severe comorbidity exists when both Internet gambling and gaming problems were present;
- Clinical studies rely on self-seeking treatment and tailored interventions.

Three studies (15.8%) addressed mainly Internet Gambling Disorder in clinical samples, suggesting it is a different clinical entity in comparison to IGD, although both share some sociodemographic characteristics (e.g., both usually affect males) and psychological features (e.g., type of emotional distress, higher harm avoidance, and reward dependence traits).

The measures used to assess gambling addiction can be considered traditional (i.e., Stinchfield’s Diagnostic Questionnaire for Pathological Gambling [49], and the Problem Gambling Severity Index (PGSI [50])).

In addition, when gambling was the main addiction and the patient played videogames, the comorbidity was more severe than for Internet gaming addiction itself [31,34,36], specifically if gaming addiction was identified together with gambling addiction (in which case paranoid ideation, distress, OCD, and interpersonal sensitivity were also present [31]). However, inversely, comorbidity did not appear in gamblers who were not gaming addicts, although the reviewed research indicated that gambling addiction appeared to be the more severe behavioral addiction. In other words, both disorders appear to be independent of each other, which is supported by evidence regarding their different clinical profiles [24]. Internet gambling had a higher mean age of disordered onset, disorder severity, somatization and depression symptoms, among other personality traits (i.e., novelty seeking and persistence) and associations with substance use (e.g., tobacco use). Furthermore, patients with both problems are younger, present more dysfunctional personality traits (e.g., lower self-directedness and higher persistence), and general psychopathology (e.g., depression, anxiety, and social phobia), higher body mass index (BMI) and food addiction (FA). In summary, although both addictive online behaviors share some emotional distress and personality traits, gambling disorder appeared to be more severe in the included studies.

Moreover, online interventions seem only to be effective when the gambler seeks treatment, and a commitment with a health professional is made, even if it is short-lived; inversely, if there is no help-seeking the efficacy of any intervention is counter-productive or may have an aversive effect [33]; therefore, ‘more is not always better’ in terms of prevention. Lastly, CBT should also be personalized to the type of gambling activity (e.g., online poker), which again requires knowledge from the therapist, as highlighted in the case of gaming [38].

### 3.4. European Policy Options to Prevent Internet Addiction Problems

**Figure 2:**
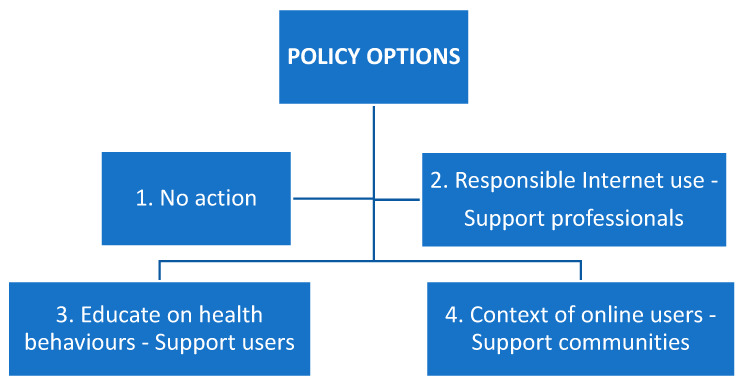
Policy options derived from the present literature review.

From the present European literature review encompassing the period between 2013 and 2018, the following four policy options have been developed based on the included 19 studies (see Figure 2).

#### 3.4.1. No Action

The first policy option concerns all stakeholders involved and should be considered with caution. In a few studies, contradictory findings have emerged which have highlighted it is not worth taking action if treatment is not sought [33] or if the problem does not emerge [30]. Thus, the first policy option is related to the following preventive actions:

- Internet addiction, Internet gaming, and gambling addictions can be hazardous (e.g., they can appear temporarily and last less than a year and then disappear). These respective problems are associated with a variety of internal and individual factors and are not always associated with the same comorbidity or associated symptom experiences. Environmental factors may also contribute to the emergence of different online addictions. Thus, Internet use-related addiction problems can appear temporarily and disappear spontaneously before they can be considered a disorder.
- This option should probably be considered when the period of the problem affecting users is less than a year, with no comorbidity or other associated severe symptoms and problems. However, if there is a suspicion of chronification (e.g., with a length of at least six months), preventive measures should be taken to avoid the development of the disorder and future disorder diagnosis.
- At present, this option seems to be unsustainable because the public health concern is growing and health practitioners are reporting increasing numbers of cases (e.g., more clinical papers than non-clinical) and interventions.

This option refers to natural recovery, which seems to exist but has not been studied much. However, due to the precautionary principle (i.e., which encourages policies that protect human health and the environment in the face of uncertain risks) it may jeopardize Europe’s ability to prevent Internet use-related problems from emerging in the first place, and to take advantage of the instruments, therapeutic, and of preventive opportunities that the scientific literature on generalized and specific Internet addiction problems has recently provided.

#### 3.4.2. Promote and Disseminate Applied Research on Responsible Internet Use and Prevention

The second policy option mainly addresses support organizations, professionals, and practitioners. Applied research is present in almost all reviewed studies, as research seeks to find solutions to the problems in European community and clinical settings. Only a couple of studies described models of GIA [25] and Internet gaming addiction [24] to provide an understanding about which factors contribute to the problems, their phenomenology, and therapy components to clinically address them. Moreover, providing information and interventions seem to be the key public health strategies to prevent and address the problem in all settings. Early preventive actions can:

- Promote evidence-based information and tools and are supported by applied research outcomes for professionals who can help to prevent and intervene in Internet use-related addiction problems. For instance, information can be available through an EU webpage with public resources (e.g., containing validated scales which exist for non-clinical measures), contact details for support organizations across the respective European countries, a list of experts, and information provided in the different EU languages.
- Include other scientific European initiatives disseminating the state of the art of research into these problems across Europe, especially if these technological problems have associated risks, such as comorbidity (e.g., anxiety and depression) and other associated psychosocial problems (e.g., cyberbullying).
- When these problems are present, self-screen tools and other test actions can be offered as a package to different professional groups and settings (e.g., clinicians in hospitals and teachers in schools), especially to those who are close to and/or work with children and adolescent populations.

There is a need to improve this research field to diversify the methodologies used and translate them to the professional sector, and to promote joint clinical and educational research. It is also crucial for standardized measures to assess problems and compare them in order to support diagnosis and treatment success (e.g., cross-culturally and trans-diagnostically). These preventive actions can provide support for decision-makers to better understand the problems from a European public health perspective, and to promote responsible Internet use and media literacy.

#### 3.4.3. Promote Education on Offline and Online Health Behaviors in Young Populations

The third policy option addresses all Internet users, especially those who have appeared as more at risk for developing Internet use-related addiction problems. All reviewed studies have highlighted common aspects related to promoting healthy Internet use, especially in adolescents and young adults, as some gaming genres are very demanding regarding competition and social involvement (e.g., MMORPGs). The following options are preventive actions which can be introduced:

- To encourage alternative motivations, engagement in alternative entertainment behaviors (also those including Internet use), new coping skills, cognitive and emotional skills, healthy attachment styles to reduce the risk of Internet use-related problems and to reduce Internet usage, if needed (e.g., through alerts and notifications) and to provide alternative options of relaxation (e.g., reading, meeting people, and engaging in physical activities).
- To detect the risk of experiencing other comorbidities or problems and address them with professional support and the support of significant others (e.g., caregivers in the case of adolescents), and to embrace systemic approaches.
- If a problem with Internet usage is present, all Internet use-related addictions should be simultaneously assessed, as many of the reviewed studies were clinical studies on gaming addiction, which together with gambling in young adults seemed to indicate the worst-case scenario regarding these problems.

In the EU, there is a need for programs and campaigns addressing children and youth to promote awareness of the risks of online behaviors at an individual person’s level. Studies have shown school interventions are usually effective, even more so if they include a psychological component (e.g., impulsivity management techniques when gaming). Young individuals should be engaged in conversations and activities concerning offline and online health, potential positive and negative implications of excessive online behaviors, and provided with information on alternative pastime activities and alternative coping strategies not involving Internet use.

The usual problem user is an adolescent who increasingly spends time gaming alone at home, usually plays MMORPGs, and experiences co-occurring problems and negative impacts in their daily life. Thus, problem cases should be treated on a case-by-case basis when detected with tailored psychological and educational interventions, including CBT, whilst ensuring treatment encompasses interventions for comorbidities. The young user should be able to determine which functions the maladaptive Internet use fulfils in his or her life, and which other options are available to him or her with professional support. These actions can be supported by other initiatives in educational and health settings, which require resources and action plans (e.g., providing funding and resources).

#### 3.4.4. Support Communities and Significant Others of Problematic Internet Users

In addition to supporting professionals (i.e., the second policy option) and users (i.e., the third policy option), communities (i.e., the fourth policy option) also need attention. The preventive actions for this group are the following:

- Enhanced family, partner, and peer communication and caretaking (e.g., through parents, siblings, partners or friends) can prevent the problems from emerging when risk indicators appear and develop progressively (e.g., excessive time spent playing online games, lack of sleep due to constant online connection, irritability and mood changes when disconnecting, neglecting school or relationships). Thus, ‘keeping an eye’ on time spent and having conversations about online uses can be the first measure of prevention in families and friendship groups.
- Information should also be available for users’ environments (e.g., for families, schools and communities) about the risks of habitually engaging in online role-playing games or online gambling applications which can be out of control and cause negative health consequences (i.e., functional impairment and distress) or financial problems (i.e., online gambling).

These problems usually affect families, education or workplace organizations, and communities. Thus, basic information, education, social, and clinical support can help individuals in the immediate context of problem users with community support. The EU should consider facilitating information provision to healthcare providers to support general practitioners when taking care of communities in health settings. Moreover, the implementation of actions, programs, and services for information, early detection, and facilitation of support and treatment routes for future problem users and their significant others are options to develop. For instance, at a school and community level, actions to promote prevention can be provided together with those for other related problems (e.g., substance use disorders and cyberbullying).

## 4. Discussion

This timely European literature review provides an overview of the currently available research on Internet use-related addiction problems in this region in the period between gaming addiction recognition by the APA and the WHO (i.e., April 2013–April 2018). It has used a public health approach and a preventative perspective to offer a set of policy options and preventive actions. The aims, therefore, were to use a cross-cultural approach across the EU to identify the problematic users’ profiles for risk management in community and clinical settings; to ascertain how Internet addiction problems have been researched in Europe within the period when gaming disorder was officially recognized by health organizations; to understand the scope of their harm implications; and, at a public health level, which preventive actions can be extracted and policy options proposed.

An update of these problems at an individual level in the EU has been provided as Internet use-related addiction problems seem to have increased world-wide in the past two decades [6,18,19], with an estimated global prevalence of 6% [18]. Low rates have generally been reported in European regions in school community sample studies (e.g., via meta-analyses and cross-cultural studies) published between 2012 and 2015 (with an average of a 2.5% prevalence [18,19,20,21]). However, this review has highlighted the prevalence is growing, as GIA in similar adolescent and young community samples is now approximately 4%–10% [23,29]. Indeed, a recent cross-cultural study has indicated that the prevalence of PIU in Europe is relatively higher than previously indicated, although this observation is based on an adult community sample (i.e., where prevalence rates ranged from 14% to 55% [51]). However, caution is needed to be considered a cause for concern in the present general population due to the highlighted conceptual and methodological issues in the respective studies of these addiction problems.

On the other hand, another indicator that requires attention and has appeared in this review is the higher rates of GIA and online gaming addiction in European clinical samples [36,37,40] compared with community samples [23,29], which range between 69%–91% for both addiction problems. This is in line with Carbonell et al.’s [52] and Lopez-Fernandez’s [53] bibliometric studies of IA and other specific online addiction problems published in the last two decades. It seems increasing world-wide Internet use is accompanied by an increasing number of publications on Internet addiction problems. However, it also seems clear gaming addiction has surpassed IA, probably due to IGD recognition by the APA [5] within the period studied, which was the starting point for this review and attracted the attention of clinicians and researchers who deal with these health problems and have published their findings since. The fact that the number of publications on gaming disorders in Europe, and internationally, is increasing may be due to the official recognition of gaming addiction as a disorder, which is indicated by the number of recent reviews [54,55,56,57,58,59]. Furthermore, neurological functions have commonalities and differences across these two behavioral addictions [54].

The number of studies included in this literature review, however, is scarce compared to other previous international reviews on GIA and IGD [5,6,16,21,54,55,56,57,58]. One explanation is that these health concerns are less prevalent in Europe relative to Asian regions, which is supported by the scientific literature. However, this does not mean that the precautionary principle cannot be applied [10], and almost no reviews have analyzed papers cross-culturally using different languages [6]. In the reviewed European samples, no study from Eastern Europe has been identified, and the regions that have seen more publications are both Southern and Western Europe for GIA, and Northern regions for gaming addiction. This is consistent with a previous cross-cultural study on dependent mobile phone use [60]. In that case, the Northern and Southern regions were the ones with heaviest online mobile use (the Northern countries especially for gaming), and the Eastern regions had lower rates. France appeared as one of the countries with the highest problematic mobile phone use, although Spain has seen a larger number of publications on Internet use-related addiction problems in the period studied.

The European scientific evidence reviewed here published between April 2013–April 2018 identified three potential problems: GIA, online gaming, and gambling disorders at community and clinical levels, which usually affected adolescents and young male adults, except for online gambling (middle-aged adults). This distribution corresponds to previous literature [5,6,16,54,55,56,57], specifically as gambling requires financial resources. However, recent empirical European and international studies on IA and gaming addiction show that females are increasingly affected, although they have not been the main study group yet [51,61]. To the best of the authors’ knowledge, no review on these problems at an individual person level in the EU has been published yet, and the main findings correspond to the results presented in two previous international reviews on clinical issues related to IA [6,55].

Furthermore, comorbidity seems to be the norm [6,59], and usually includes depression, social anxiety disorder, social phobia, OCD, ADHD, hostility, substance use disorders (e.g., gambling, alcohol, marihuana, nicotine, and cocaine use), eating disorders (e.g., binge eating disorder, bulimia, and obesity), and certain personality traits and personality disorders (e.g., impulsivity, borderline, avoidant personality, or antisocial disorders) [6,55]. However, the present review showed different comorbidities depending on the type of Internet use-related addiction problems, a novel finding which reinforces the independent identities of GIA and gaming addiction [5,24,38,53,54,55,56,57,58,59]. In GIA, half of the investigated samples present with comorbid Axis I disorders, which is consistent with previous research [25,27,62]. This suggests a complete psychiatric evaluation is needed for these types of problems. Nevertheless, this review also highlights the need of a psychological evaluation as other emotional, cognitive, and behavioral features have emerged, such as the role of self-esteem [25,32,39], attachment or defense styles [26,60], cognitive coping and disassociation [25,32], and other personality traits and mental disorders [6,25,27,37,41,59] in specific developmental stages (i.e., adolescence [58]). In gaming addiction, however, the spectrum of comorbid disorders is more diverse and severe, especially if gambling is one of the co-occurring disorders, including internalizing and externalizing profiles which need to be considered regarding recovery length [24,35,36,38,39,59]. A European gamer profile has also emerged where environmental factors appear for the etiology, development, and recovery of these problems (e.g., CBT with a systemic approach for adolescent gamers).

At present, research has moved the field forward considerably, resulting in clinicians and researchers recognizing Internet use-related addiction problems across different devices [30,53,60,62] as more scientific research is emerging [5,6,52], and so is the demand for diagnosis and treatment [24,34,36,38,39,41]. The present literature is slightly contradictory, as it has been suggested that the device used to engage in gaming can be associated with the occurrence, course, and prognosis of IGD [62], and it has also been stated that the device does not influence gaming addiction problems [30]. The most alarming studies on gaming addiction and the role of gaming devices come from Asia, and those studies which contradict the findings related to the role of gaming devices in gaming addiction were mainly conducted in Europe.

On the other hand, other contradictions regarding gaming addiction, such as comorbidity or associated symptom experience identified, are the reasons why, together with the low number of prevention research studies in the field [13,16,57,59,63], it seems essential to start addressing possible preventive actions regarding IA and related harms at all levels (i.e., by regions and globally). Simultaneously, qualitative work is needed to address the uniqueness of the phenomenological expression of these types of behavioral addiction problems [64] facilitated through the Internet.

Regarding the policy options, the first one (i.e., no action) has also been put forward in similar preventive studies on substance use disorders. This action is based on the available knowledge on natural recovery, which is considered in the context of IGD as well [65]. However, as almost no follow-up studies have produced evidence on long-term relapse and the natural recovery rates, caution must be applied for this first policy option. The second policy option will aid informing and training professionals and practitioners and is aligned with the few international reviews on IA and prevention. For instance, according to Vondráčková and Gabrhelík [16], the improvement of skills in specific professions (i.e., for researchers, counsellors, and teachers) can support preventive action and better intervention plans or, as Kiraly et al. [57] stated, measures are taken to make health services available to gamers who experience problems. The third policy option, related to users, also highlights the need to pay attention to those who are among the highest risk groups, individuals of particular age (e.g., children and youth, especially MMORPG gamers), gender (males), engaged in a general or specific Internet activity, and who experience comorbidity [6,15,16,55,57,58,59]. The fourth policy option is aimed at supporting communities, including signposting families, schools, institutions, and governments [13,16,57,63,66,67].

Nevertheless, as King et al. [13] highlighted, in Western cultures including Europe, at the moment community-based support derives from non-profit organizations and the private sector; although a few countries are starting to provide support through their national health systems, such as Germany [10]. However, not all measures that have been put into place to prevent Internet and gaming addiction have obtained effective results [13,57], and there is a bias regarding what is known through the current literature, which is dominated by English language publications from English and Asian regions [13,57]. Thus, the current scientific literature base may not sufficiently reflect what other non-English speaking countries are already doing regarding prevention at all levels (e.g., Switzerland [10]).

This review also has its limitations, including the strategy applied to identify the included studies. For instance, the period of five years selected, and the number of databases used can be considered short and small, but both decisions have a rationale (i.e., recognition of gaming addiction, and disciplinary and interdisciplinary scientific search engines associated with the aims, respectively). Preliminary findings are relevant as they have shown, for example, the emergence of clinical research in online addiction problems in Europe with its specificities (e.g., gamer profile and specific comorbidity depending on internet use-related problems). However, in this emergent field, other literature reviews have also been undertaken with even shorter periods of analysis for relevant reasons and with a larger or smaller number of databases (e.g., internet use-related to self-harm and suicidal behaviour using Medline, Cochrane, and PsychINFO, and covering four years [68], or the utility of magnetic resonance imaging to study IA using Scopus, which covered a three year period [69]). The keywords applied did not take into consideration other possible Internet addiction problems which are currently being researched in Europe (e.g., cybersex). However, the identified limited number of studies produced preliminary findings to achieve the present aims to obtain an overview of the status quo in Europe regarding these problems from a cross-cultural and preventive perspectives, with a qualitative analysis using the lens of harm minimization to develop a set of policy options with preventive actions. Future research should first extend a similar procedure to non-European countries and also collect grey literature with non-English language publications to produce a holistic perspective of the policy options and prevention actions and consequences of what kinds of initiatives are already taken in several countries, which can be useful at local and global levels. Thus, this review offers a brief and timely snapshot of scientific studies in the recent period where gaming addiction has been officially recognized. Indeed, it is the first review on these problems at a European level using a preventive approach. However, methodological improvements can aid more robust future research, which should apply methods and procedures to compute other quantitative inter-rater reliability measures to complement the qualitative inter-rated reliability obtained through sharing and comparing coding agreements in iterative rounds until arriving at a consensus and theoretical saturation of findings (e.g., the Cohen’s Kappa coefficient [70]), and complementary quality checks of the procedure (e.g., the Critical Appraisal Skills Programme (CASP) [71]). The included studies’ findings have been synthesized and analyzed in detail in the present literature review to provide an overview regarding these emerging addiction problems in Europe, which can be used with caution as the present literature review constitutes a qualitative narrative synthesis, for international comparisons, and to translate some of the identified policy options into preventive actions.

## 5. Conclusions

In summary, the most prevalent Internet addiction problems appeared to be generalized Internet addiction and online gaming addiction in the EU between April 2013 and April 2018, both of which tend to present with specific comorbid disorders. More clinical studies compared to non-clinical studies were identified and analyzed which shows the emergence of and need for action, public health, and prevention. Gaming and gambling addictions were usually more severe problems compared to generalized Internet addiction. In addition, gambling appears to be more severe than gaming. However, the current scientific literature base does not report much prevention work in Europe (and internationally). A set of preventive recommendations and policy options have been formulated, which can support future harm minimization actions.

## Figures and Tables

**Table 1:** Papers selected for the review (*N* = 19).

Authors (Year) [Reference]	Country	Problem
Andrisano, Santoro, De Caro, Palmieri, Capunzo, Venuleo, & Boccia (2016) [23]	Italy	Internet addiction
Beranuy, Carbonell, & Griffiths (2013) [24]	Spain	Gaming addiction
Brand, Laier, & Young (2014) [25]	Germany	Internet addiction
Danet, & Miljkovitch (2016) [26]	France	Internet addiction
Floros, Siomos, Stogniannidou, Giouzepas, & Garyfallos (2014) [27]	Greece	Internet addiction
Frölich, Lehmkuhl, Orawa, BrombaWolf, & Görtz-Dorten (2016) [28]	Germany	Gaming addiction
González, & Orgaz (2014) [29]	Spain	Internet addiction
Holstein, Pedersen, Bendtsen, Madsen, Meilstrup, Nielsen, & Rasmussen (2014) [30]	Denmark	Gaming addiction
Jiménez-Murcia, Fernández-Aranda, Granero, Chóliz, La Verde, Aguglia, & del Pino-Gutiérrez (2014) [31]	Spain	Gambling & Gaming addictions
Lai, Altavilla, Mazza, Scappaticci, Tambelli, Aceto, & Tonioni (2017) [32]	Italy	Internet addiction
Luquiens, Tanguy, Lagadec, Benyamina, Aubin, & Reynaud (2016) [33]	France	Gambling addiction
Mallorquí-Bagué, Fernández-Aranda, Lozano-Madrid, Granero, Mestre-Bach, Baño, & Jiménez-Murcia (2017) [34]	Spain	Gambling & Gaming addictions
Marco, & Chóliz (2017) [35]	Spain	Gaming addiction
Martín-Fernández, Matalí, García-Sánchez, Pardo, Lleras, Castellano-Tejedor (2017) [36]	Spain	Gaming addiction
Müller, Beutel, & Wölfling (2014) [37]	Germany	Internet addiction
Taquet, & Hautekeete (2013) [38]	France	Gaming addiction
Torres-Rodríguez, Griffiths, Carbonell, Farriols-Hernando, & Torres-Jimenez (2017) [39]	Spain	Gaming addiction
Van Rooij, Schoenmaker, & van de Mheen (2017) [40]	The Netherlands	Gaming addiction
Wölfling, Beutel, Dreier, & Müller (2014) [41]	Germany	Internet addiction & Gaming addiction
